# Recombinant luciferase-expressing human cytomegalovirus (CMV) for evaluation of CMV inhibitors

**DOI:** 10.1186/1743-422X-8-40

**Published:** 2011-01-26

**Authors:** Ran He, Gordon Sandford, Gary S Hayward, William H Burns, Gary H Posner, Michael Forman, Ravit Arav-Boger

**Affiliations:** 1 Department of Pediatrics, Johns Hopkins University School of Medicine, Baltimore, MD, USA; 2 The Sidney Kimmel Comprehensive Cancer Center, Johns Hopkins University School of Medicine, Baltimore, MD, USA; 3 Department of Chemistry, School of Arts and Sciences, The Johns Hopkins University, Baltimore, MD, USA; 4 Department of Pathology, Johns Hopkins Medical Institutions, Baltimore, MD, USA

## Abstract

Recombinant Towne CMV expressing luciferase under the control of CMV-DNA polymerase (*POL*) or the late pp28 (UL99) promoters were evaluated for potential application in high-throughput screening of anti-viral compounds. *POL*-and pp28-luciferase displayed maximal expression 48 and 72 hours post infection, respectively. The pp28-luciferase virus achieved a wider dynamic range of luciferase expression (6-7 logs) and was selected for testing of inhibition by five anti-viral compounds. Luciferase expression highly correlated with plaque reduction and real-time PCR. The pp28-luciferase reporter system is rapid, reproducible, and highly sensitive. It may be applied to screening of novel anti-CMV compounds.

## Background

Infection with Cytomegalovirus (CMV) continues to be a major threat in organ transplant recipients and congenitally-infected children [1,2]. Although existing systemic therapies are effective in suppressing virus replication, serious side effects and the emergence of resistant viral strains pose significant treatment dilemmas [3]. The need to identify and develop new anti-CMV compounds coincides with the advancement of rapid, sensitive, and high-throughput methods for screening of lead compounds. While the plaque reduction assay remains the gold-standard for screening of anti-viral compounds, new techniques based on recombinant DNA technology and highly sensitive molecular assays have recently been suggested as alternatives [4-6]. Real-time PCR, the standard of care in the management of CMV disease in high- risk patient populations, may also provide a sensitive tool for drug screening [7-12]

In earlier studies, a series of chloramphenicol acetyl transferase (CAT) recombinants expressing CAT under the control of UL54 (DNA polymerase, *POL*) or UL99 (pp28) promoters were constructed. The expression of CAT in infected cells highly mimicked the expression pattern of the endogenous UL54 and UL99 [4,13]. Thus, these two gene promoters were selected to construct luciferase-recombinant CMV for quatification of CMV replication in a rapid and reproducuble way. We report on the evaluation of two luciferase recombinant viruses (pp28 and *POL*) and the correlation of the pp28-luciferase system with plaque reduction and real-time PCR in evaluation of CMV inhibition by anti-CMV compounds.

## Methods

### Construction of luciferase viruses

Recombinant CMV based on the laboratory-adapted strain, Towne, was constructed by homologous recombination in transfected-infected cells. A β- galactosidase (β -gal)-expressing Towne virus was first constructed using an intergenic insertion site between US9 and US10. Prior studies in which a β-glucuronidase expression cassette was inserted in this intergenic region of the laboratory-adapted AD169 virus revealed no alteration in expected transcription from this region [4,14,15]. The recombinant was genetically stable and exhibited normal *in-vitro *growth characteristics. The transfer vector, pT, was constructed from pRL120 which contains the Towne virus HindIII T fragment [16]. A 2.0 kb BamHI-ApaI subfragment containing US9 was ligated into pGEM11z (Promega, Madison, WI) and the adjacent 1.3 kb ApaI-ApaI fragment containing US10 was isolated from agarose gels and ligated into the ApaI site. DNA sequencing confirmed the correct orientation of this fragment. The BstEII site, which lies midway between the US9 and US10 genes, was used as the insertion site for the β-gal expression cassette containing an SV40 promoter and polyA signal (pSVβ from Clontech, Mountain View, CA). DNA extracted from human foreskin fibroblasts (HFF) infected with Towne virus and linearized transfer vector containing the expression cassette were coprecipitated onto subconfluent HFF cultures by the calcium phosphate method [17], followed by a 2 min shock with 20% Dimethyl sulfoxide (DMSO) in Minimum Essential Medium (MEM) 4 to 6 hrs later. Virus from cultures developing cytopathic effects was passed onto fresh HFF cultures, and examined for β -galactosidase activity. Recombinant virus, designated T242, was isolated from positive cultures by limiting dilution in 96 well plates of HFF and selection of β-gal positive wells at the highest dilutions.

To produce a recombinant virus expressing the luciferase reporter gene under the control of either the promoter of an early gene (*POL*, UL54) or a late gene (pp28, UL99), the expression cassette of luciferase was substituted for the β-gal cassette using the same transfer vector (pT). Expression cassettes of luciferase under the control of *POL*- or pp28-promoter were constructed by cloning the PCR products of the upstream 500 bp of DNA polymerase or 350 bp of pp28 genes and ligating them into the 5' position of the luciferase coding region. These expression cassettes were then ligated into the blunted BstEII site of the pT transfer vector, linearized and used in coprecipitation experiments with the DNA of HFF cells infected with T242. Successful replacement of the β-gal expression cassette by the luciferase expression cassettes with loss of β-gal expression and acquisition of luciferase expression as phenotypic markers facilitated isolation of the desired recombinants. Several PCR sequencing reactions confirmed the correct position and orientation of the luciferase reporter gene. The following primers were used: primer 1- US09 forward 5'-ACCTTGAAATGGGTCGCGCTCCGCT-3', primer 2- luciferase forward-5'-ACAAGGATATGGGCTCACTGAGACT-3', primer 3: luciferase reverse 5'-AGTCTCAGTGAGCCCATATCCTTGT-3', and primer 4- US10 reverse- 5'-GCTATCGTCGCCGGAAGGAAACCGA -3'.

### Cell Culture and virus infection

HFF and human lung fibroblasts (HEL) (ATCC, CRL-2088 and CCL-137, respectively) were propagated in Dulbecco's Modified Eagle Medium (DMEM) containing 10% fetal bovine serum (FBS) and used for infections with the luciferase viruses. For assays other than plaque reduction, 4 × 10^4 ^HFF cells were seeded in each well of 24-well plate one day prior to infection. Luciferase viruses were used for infections with multiplicity of infection (MOI) of 1.0 as previously described [18]. After 90 minutes adsorption, virus was removed, and 0.5 ml of media containing specified concentrations of antiviral compounds was added. Infected non-treated cells were used as positive controls; non-infected cell lysates were used as negative controls.

### Luciferase Assay

HFF cells were collected and lysed with Wizard^® ^SV Lysis Buffer (Promega, Madison, WI). The lysates were assayed for luciferase and cell viability using an automated luminescent assay (Promega, Madison, WI), and CellTiter-Glo luminescent cell viability assay kit, respectively, on GloMax^®^-Multi+ Detection System (Promega, Madison, WI) according to manufacturer's instructions.

### Plaque reduction assay

HEL cells were seeded at 3 × 10^5 ^cells per well in twelve-well plates and were infected 24 hours later with the pp28-luciferase CMV at 60 PFU/well. Following 90 minutes adsorption, the medium was aspirated from the wells, and fresh medium containing selected drug dilutions of ganciclovir (GCV), Foscarnet (FOS), Cycloheximide (CHX), artesunate (ART), dimer sulfone carbamate [19] and 0.5% of carboxymethyl-cellulose were added into triplicate wells. After incubation at 37°C for 8 days, the overlay was removed, and the monolayer was stained with crystal violet. Plaques were counted microscopically under low power (40×). Drug effects were calculated as the percentage of reduction in number of plaques in the presence of each drug concentration to the number observed in the absence of drug.

### Virus yield reduction assay

HFF were infected with the original Towne virus or pp28/*POL*-- luciferase virus at an MOI of 0.1. Culture supernatants were collected every two days until day 10 post infection and frozen at -80°C. Collected samples were thawed and used for titration of infectious virus by the plaque assay.

### Real-time PCR

The quantitative CMV real-time PCR assay is based on detection of a 151bp region from the highly conserved US17 gene [20]. The limit of detection of the assay is 100 copies/mL (3.0 copies/reaction), and the measureable range is 2.4-8.0 log_10 _copies/mL. The PCR was performed using a total reaction volume 50 μL. This included 25 μL of TaqMan 2X Universal PCR Master Mix (Applied Biosystems, Foster City, CA), 1.5 μL each of 10 μM primers, 1 μL of 10 μM FAM-labeled probe, 11 μL of dH_2_0, and 10 μl of template. Amplification was performed on a 7500 Real-Time PCR System (Applied Biosystems, Foster City, CA). PCR conditions were: 50°C for 2 min, 95°C for 10 min, 40 cycles of 95°C for 15 s and 60°C for 60 s. Quantification standards were prepared by cloning the US17 amplicon in the pCR^®^2.1-TOPO^® ^plasmid vector (Invitrogen, Carlsbad, CA). Serial 10-fold dilutions of plasmid from 7.0 to 1.0 log_10 _copies/reaction were included with each assay and used to establish a standard curve. Assay controls included quantified CMV AD169 DNA (Advanced Biotechnologies Inc.) and quantified Towne CMV at 3.0 and 5.0 log_10 _copies/mL. Quantitative CMV data were expressed as viral DNA copies per milliliter.

### Antiviral compounds

GCV, sodium phosphonoformate (FOS) and cycloheximide (CHX) were obtained from Sigma-Aldrich (St. Louis, MO). Artemisinin derivatives, monomeric trioxane artesunate (ART) and trioxane dimer sulfone carbamate were synthesized at Johns Hopkins University (GHP), and their structural details have been provided elsewhere [18].

## Results

### Luciferase constructs

**Figure 1:**
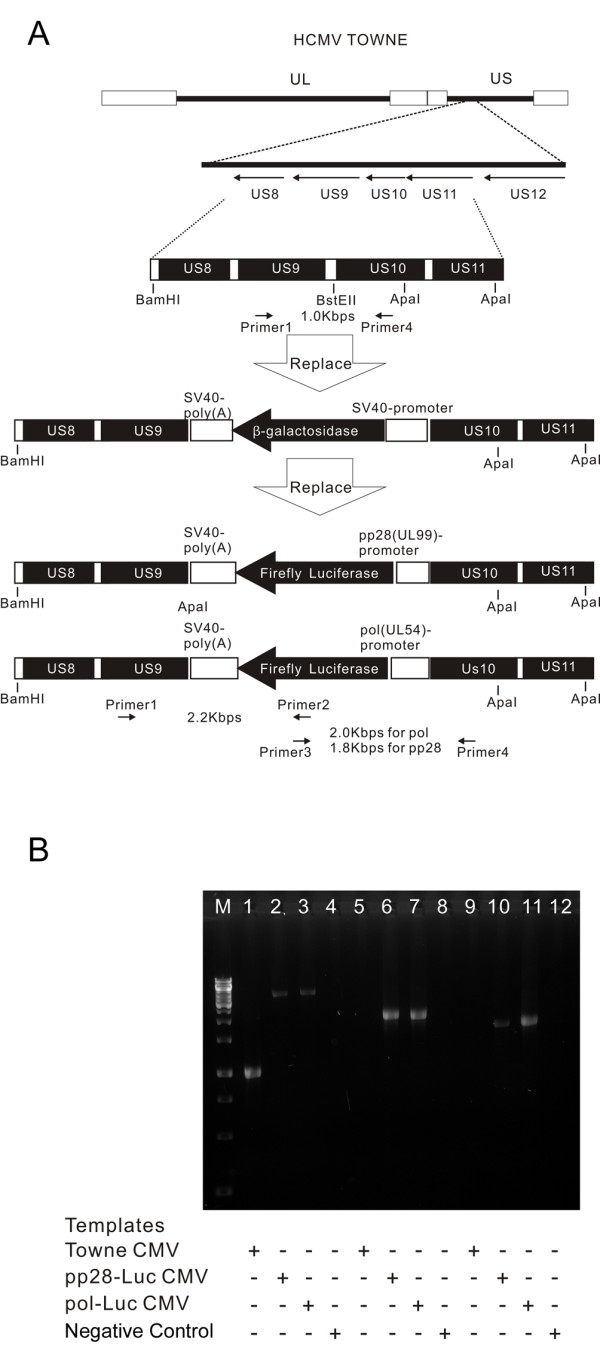
**Construction of luciferase-recombinant CMV viruses and confirmation of luciferase orientation by PCR**. 1(A): Construction of luciferase-recombinant Towne, insertion of promoter and luciferase reporter between US9 and US10. Appropriate restriction sites, the primers used for verification and the expected size of PCR products are depicted. 1(B): PCR of pp28- and *POL*-luciferase constructs. Lane 1-4: primers 1+ 4, lane 5-8: primers 1+2, lane 9-12: primer 3+4.

Two luciferase expressing viruses were constructed with the Towne CMV strain (Figure 1A). A recombinant β-galactosidase (β-gal) CMV strain was first prepared as a backbone for luciferase CMV. Recombinant β-gal virus was isolated from positive cultures. This virus was used in a second-round DNA recombination to generate two luciferase-reporter CMV viruses: the luciferase gene being under the control of either UL54 (*POL*) or UL99 (pp28) promoters. Successful recombinants were isolated by loss of β-gal activity and the expression of luciferase protein. The loss of the β-gal gene and acquisition of the luciferase gene in the expected location was confirmed by DNA sequencing (Genebank submission ID: 1420040, sequences are also available in Additional file 1). Insertion at the specific sites was verified by PCR sequencing (Figure 1B).

### Comparison of luciferase expression by the two viral constructs

**Figure 2:**
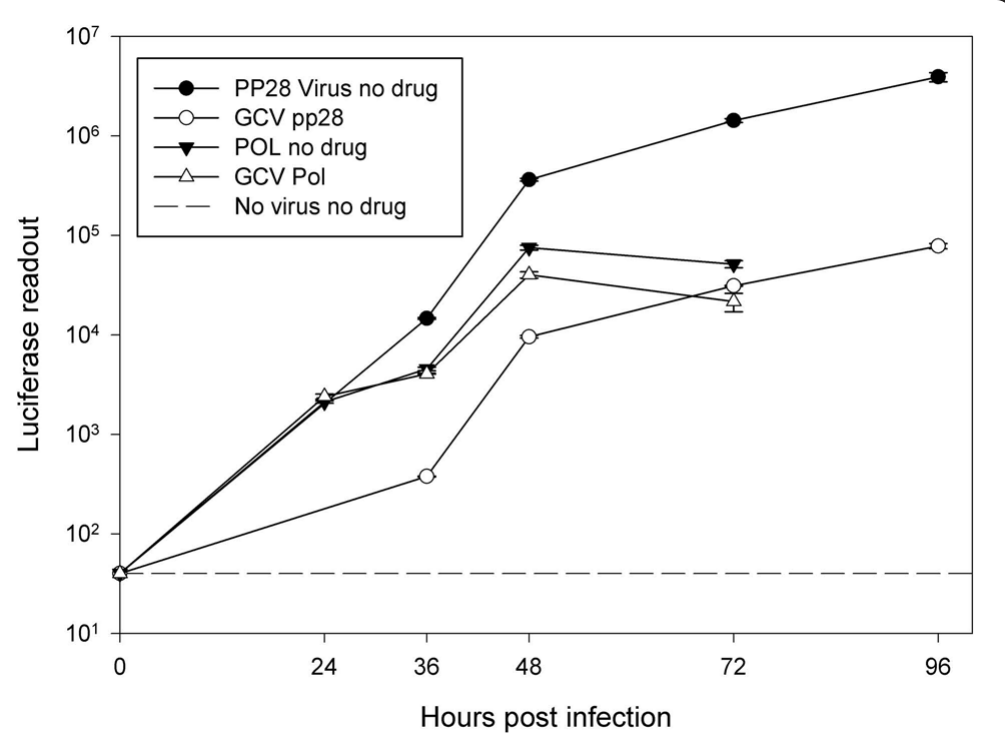
**Timing and expression pattern of pp28-and *POL*-luciferase CMV**. Luciferase expression was determined in cell-lysates at indicated time points following infection with pp28- or *POL*-luciferase with and without treatment with GCV (30 μM). Y axis-log scale of luciferase read out; X axis- time points in hours.

The recombinant viruses were expected to express luciferase at different stages of virus replication. The early gene UL54 (*POL*) is expressed within the first 24 hours post infection (hpi), usually later than 12 hpi [21]; whereas the true late UL99 (pp28) gene is expressed only at or after 48 hpi. Luciferase expression by *POL*- and pp28-luciferase was quantified in cell lysates at 12, 24, 36, 48, 72 hpi, and at 36, 48, 72 and 96 hpi, respectively (Figure 2). Using the same cell conditions, infectivity, and luciferase assay system, peak luciferase activities measured with pp28-luciferase were 20 fold higher than those measured with *POL*-luciferase. The peak activity of pp28-luciferase was reached at 72 hpi, followed by a plateau towards 96 hpi. *POL*-luciferase reached its maximum expression at 48 hours post infection. The dynamic range of the luciferase assay using pp28-luciferase and *POL*-luciferase was 50 - 5 × 10^6^, and 50 - 6 × 10^4 ^respectively; therefore the pp28-luciferase virus was used in subsequent experiments.

### Growth Characteristics of pp28-luciferase and the parent Towne virus

**Figure 3:**
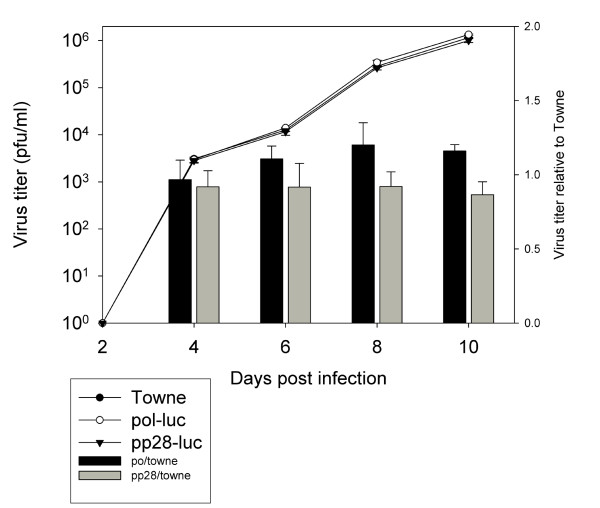
**Growth characteristics of Towne, pp28-and *POL*-luciferase Towne viruses**. The production of virus progeny was determined in HFF infected with the original Towne virus, and recombinant pp28- or *POL*-luciferase virus at an MOI of 0.1. Culture supernatants were collected at the indicated days and used for titration of infectious virus by the plaque assay. Y-axis on the left indicates growth of progeny viruses in log scale, Y-axis on the right indicated relative virus kinetics of the recombinant viruses as compared to the parent Towne strain.

We evaluated whether insertion of the recombination cassette affected the growth kinetics and production of infectious progeny. The parent Towne virus, pp28- and *pol*-luciferase Towne viruses were grown in HFF and the production of infectious progeny was determined every two days during 10 day course post infection. The growth characteristics of the viruses were similar (Figure 3). A marked increase in virus production was observed starting 2 days post infection, and growth kinetics was similar to previous reports [22]

### Correlation of plaque reduction and luciferase expression

**Figure 4:**
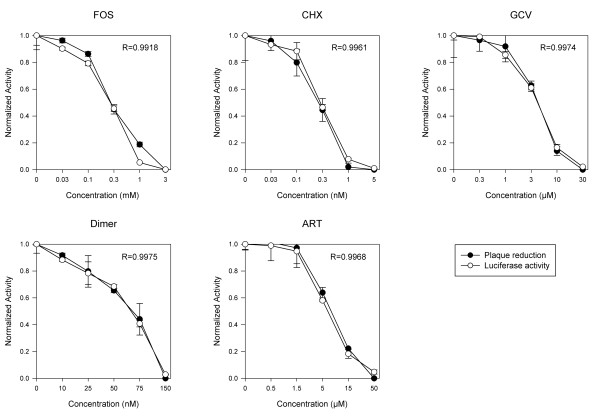
**Correlation of plaque reduction and luciferase expression**. CMV-infected HFF were treated with GCV, FOS, CHX, ART, dimer sulfone carbamate with the indicated drug concentrations. Luciferase expression was quantified in cell lysates 72 hpi. Plaque reduction was performed 10 days post infection. The correlation coefficient is provided for each experiment.

Parallel experiments were conducted using the same MOI of pp28-luciferase CMV with and without anti-CMV compounds (GCV, FOS, ART, dimer sulfone carbamate, CHX). The relative number of plaques counted 10 days post infection was compared to relative luciferase activities assayed 72 hpi (Figure 4 Table 1). The drug concentration inhibiting 50% virus replication (EC_50_) by plaque reduction and luciferase expression was determined for each compound. For all five compounds a high correlation was observed between plaque reduction and luciferase expression (Figure 4). Data obtained with the plaque reduction assay were similar to previous reports (Table 1).

**Table 1:** Inhibition of pp28-luciferase by anti-CMV compounds using plaque reduction or luciferase assay

Compound	Plaque Reduction EC_50 _(μM)	Luciferase EC_50 _(μM)	Reference
FOS	328 +/- 28	268 +/- 20	[28]
Dimer Sulfone Carbamate	0.067 +/- 0.011	0.066 +/- 0.004	[18]
ART	8.03 +/- 0.55	6.74 +/- 0.38	[29]
GCV	4.39 +/- 0.39	4.23 +/- 0.27	[30]
CHX	0.262 +/- 0.067	0.299 +/- 0.036	NA

EC_50 _was determined by plaque reduction assay or luciferase expression in pp28-luciferase CMV infected HFF cells. Reported values represent the means ± standard deviations (SD) of data derived from at least three independent experiments performed in duplicate. Historical controls are provided for EC_50 _values (reference column).

### Inhibition of luciferase expression and DNA replication by dimer sulfone carbamate and GCV

**Figure 5:**
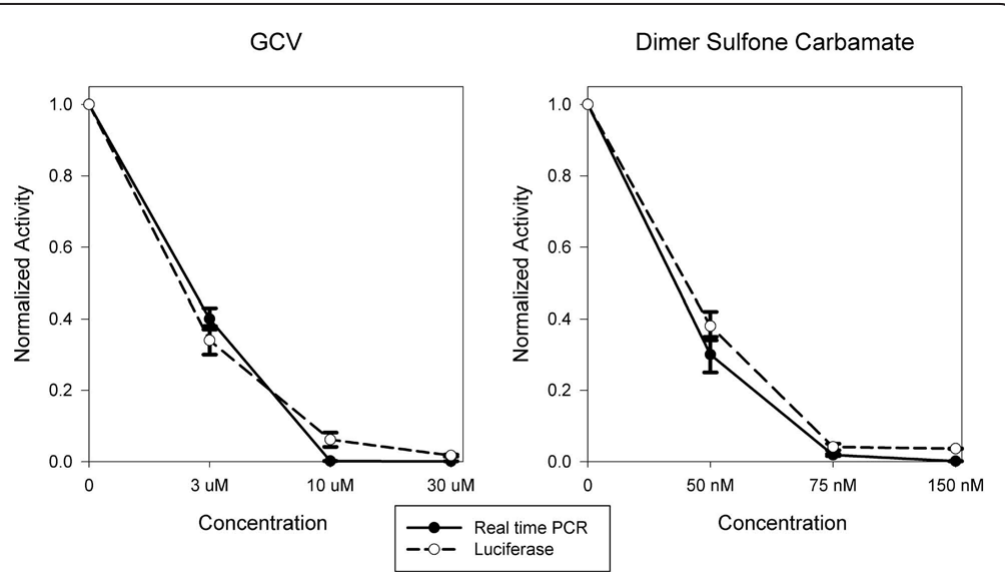
**Luciferase expression and real-time PCR**. HFF were infected with pp28-luciferase and treated with either GCV or dimer sulfone carbamate. Luciferase activity was determined in cell lysates of infected-treated cells and infected non-treated cells. DNA copy number was determined by real-time PCR in supernatants of infected-treated cells and infected non-treated cells 6 days post infection.

The supernatants from infected-treated and infected-non treated cells were used for real-time PCR at day 3. However, the test was not sensitive enough to detect differences between the treatment conditions (data not shown). Therefore, luciferase activity was compared with real-time PCR from supernatants of infected cells 6 days post infection. A high correlation was found between luciferase expression, and DNA copy number (Figure 5).

## Discussion

We report on a highly sensitive and objective luciferase reporter assay for determination of CMV inhibition by anti-viral agents. The assay, based on pp28-luciferase recombinant CMV, can be performed 72 hpi and drug treatment, has a large dynamic range of 6-7 logs, and is highly reproducible. Our work also reveals a high degree of correlation between late gene (luciferase) expression and plaque enumeration further confirming the potential use of this assay in screening of anti-viral activities.

The susceptibility of CMV strains, laboratory-adapted and clinical isolates, to anti-CMV compounds has traditionally been evaluated by the classic plaque assay [23]. Although this assay best reflects viral infectivity, or the biological behavior of CMV, it suffers from several drawbacks. The assay is time consuming; results are usually available 8-21 days after infection depending on the virus strain used, and counting of plaques is labor intensive. Another disadvantage of the plaque assay is that the amount of viral replication within a single cell cannot always be determined. Not infrequently, the endpoint of the test shows enlarged cells (CPE) without spread of the virus to adjacent cells (plaque).

Recombinant viruses carrying different reporter genes have been developed as alternative methods to overcome some of the limitations of the plaque assay. A recombinant CMV expressing β-galactosidase under the control of the major immediate early promoter was used in a 96-well assay [24]. Although the assay was sensitive and rapid, background β-galactosidase activity was observed secondary to its expression under the control of an immediate early gene during the initial infection. A secreted alkaline phosphatase (SEAP) reporter gene driven by the CMV major immediate early promoter was inserted at the US6 gene [25]. Reduction in SEAP activity under drug treatment was used to determine drug sensitivity. Results of transferring specific mutations in UL97 or *POL *were compared with results obtained using traditional phenotyping assays. The assay was validated for approved CMV drugs (GCV, FOS, and CDV) that target the CMV DNA polymerase. The open reading frame between US9 and US10 has been used to construct several recombinant CMV strains [4,5,26]. For example, a GFP- reporter system generated with the laboratory-adapted strain AD169 was applied successfully to both qualitative and semiquantitative applications [5]. Compared to the GFP-CMV system, the luciferase-CMV offers a highly accurate and quantitative assay which is simple and easy to perform. A limited evaluation of pp28 -luciferase CMV activity in the presence of GCV, acyclovir and papaverine, suggested its potential application for anti-viral screen [26].

In addition to recombinant viruses, reporter cell lines have been generated to screen for anti-CMV compounds [6,27]. In one such approach, using a luciferase reporter cell line, the promoter was activated by immediate early proteins; therefore compounds that inhibit CMV at later stages of infection cannot be evaluated with this system [6]. Since the pp28-luciferase virus is driven by the promoter of a true late CMV gene, which can only occur after DNA replication and the onset of transcription of late genes, it can be applied for screening of compounds that target steps prior to and during DNA replication. The pp28-luciferase system therefore has a much wider application for drug screening compared to the reported luciferase cell line [6].

Quantification of viral genomes by real-time PCR is generally proportional to production of virus particles [7]. Application of real-time PCR for *in-vitro *screening of antiviral compounds is attractive because the assay is rapid and highly-sensitive. However, compared to the luciferase assay, real-time PCR is more labor-intensive. DNA copy number measured in supernatants collected at 6 days post infection with Towne virus correlated with luciferase activity in cell lysates at 3 and 6 days post infection. For a clinical isolate, generally 10 days were required for quantification of DNA in cell lysates [18]. Recently, a real-time PCR assay of a conserved region in UL54 was performed in cell lysates four days following infection and treatment with compounds and showed a high correlation with plaque reduction assay [12]. Additional studies are needed to determine the best timing and compartment for performance of the real-time PCR assay.

Our study reveals late CMV protein expression highly correlates with the production of infectious progeny (plaque assay) and DNA replication. Advantages of the luciferase assay over the real-time PCR include: faster turn-around time after infection, and lower cost (20 times less than real-time PCR). The luciferase assay yielded similar data to the plaque assay, but its performance (accuracy and rapidity) was superior. In conclusion, the recombinant pp28-lucifarese fulfills important characteristics that are required for high-throughput screening of anti-viral compounds: rapidity, reproducibility, low cost, and high sensitivity.

## Abbreviations

CMV: Cytomegalovirus; PCR: polymerase chain reaction; EC_50_: effective concentration 50; HEL: human embryonic lung fibroblasts; HFF: human foreskin fibroblasts; MOI: multiplicity of infection; US: unique short; *POL*: polymerase.

## Competing interests

The authors declare that they have no competing interests.

## Authors' contributions

RH carried out the plaque/luciferase assays and verification of viral constructs. He participated in drafting the manuscript. GS, GSH and WHB designed and constructed the luciferase viruses, GHP synthesized and provided artemisinin derivatives, MF carried out the real-time PCR assays, RAB directed the study, analyzed and interpreted the data, drafted and revised the manuscript. All authors read and approved the manuscript.

## Supplementary Material

Additional file 1 **Sequences of the pp28, *POL *promoters and luciferase in the region between US9 and US10**. Several regions can be distinguished- bold sequences are of CMV Towne, underlined sequences are *POL *(sequence #1) and pp28 (sequence #2) promoters, and the *italic *regions are the sequence of firefly luciferase gene. Click here for file
