# Supplementary material for: Recombinant luciferase-expressing human cytomegalovirus (CMV) for evaluation of CMV inhibitors
Source: Virol J. 2011 Jan 26;8:40. doi: 10.1186/1743-422X-8-40 (PMC3041771; doi:10.1186/1743-422X-8-40)
Supplement: Additional file 1 — Sequences of the pp28, POL promoters and luciferase in the region between US9 and US10. Several regions can be distinguished- bold sequences are of CMV Towne, underlined sequences are POL (sequence #1) and pp28 (sequence #2) promoters, and the italic regions are the sequence of firefly luciferase gene. [file 1743-422X-8-40-S1.DOCX]

Sequence #1: *POL*-luciferase Towne

1 **gctatcgtcg ccggaaggaa accgaccccg aaactattct gttaccctaa cactcgcctc**

61 **ccgggattgt tatgaacgct tcgtgtgccc ggtatacgat tccgggacgc cgatgggcgt**

121 **tttgatgaac ttgacgtacc tctggtatct aggcgactac ggggcgatac taaaaattta**

181 **tttcggactg ttttgcgggg cctgtgtcat cacccgatcc ctcctcctga tatgtggtta**

241 **ttatccacct cgcgaataaa atgatttgtt atgatgtcat tgtttactga aaaggaatgt**

301 **gctttcccgg catgggcccg attccgagaa atggtatgat gaatcatgtg gtcaggcgct**

361 **gttctcaacg tccatataaa cgtgggtttc g**gtgacgtac cgagctcgcg gtggcctccg

421 tggtcgaagg cgttgccacc ttcctcaaaa accccttcgg agccttcacc atcatcctcg

481 tggccatagc cgtagtcatt atcacttatt tgatctatac tcgacagcgg cgtctgtgca

541 cgcagccgct gcagaacctc tttccctatc tggtgtccgc cgacgggacc accgtgacgt

601 cgggcagcac caaagacacg tcgttacagg ctccgccttc ctacgaggaa agtgtttata

661 attctggtcg caaaggaccg ggaccaccgt cgtctgatgc atccacggcg gctccgcctt

721 acaccaacga gcaggcttac cagatgcttc tggccctggc ccgtctggac gcagagcagc

781 gagcgcagca gaacggtaca gattctttgg acggacagac tggcacgcnn ncaagggaca

841 gaagcctaac ctgctagacc ggctgcgaca tcgcaaaaac ggctacagat ctaagtaagc

901 ttggcattcc cggtactgtt ggtaaa*atgg aagacgccaa aaacataaag aaaggcccgg*

961 *cgccattcta tcctctagag gatggaaccg ctggagagca actgcataag gctatgaaga*

1021 *gatacgccct ggttcctgga acaattgctt ttacagatgc acatatcgag gtgaacatca*

1081 *cgtacgcgga atacttcgaa atgtccgttc ggttggcaga agctatgaaa cgatatgggc*

1141 *tgaatacaaa tcacagaatc gtcgtatgca gtgaaaactc tcttcaattc tttatgccgg*

1201 *tgttgggcgc gttatttatc ggagttgcag ttgcgcccgc gaacgacatt tataatgaac*

1261 *gtgaattgct caacagtatg aacatttcgc agcctaccgt agtgtttgtt tccaaaaagg*

1321 *ggttgcaaaa aattttgaac gtgcaaaaaa aattaccaat aatccagaaa attattatca*

1381 *tggattctaa aacggattac cagggatttc agtcgatgta cacgttcgtc acatctcatc*

1441 *tacctcccgg ttttaatgaa tacgattttg taccagagtc ctttgatcgt gacaaaacaa*

1501 *ttgcactgat aatgaattcc tctggatcta ctgggttacc taagggtgtg gcccttccgc*

1561 *atagaactgc ctgcgtcaga ttctcgcatg ccagagatcc tatttttggc aatcaaatca*

1621 *ttccggatac tgcgatttta agtgttgttc cattccatca cggttttgga atgtttacta*

1681 *cactcggata tttgatatgt ggatttcgag tcgtcttaat gtatagattt gaagaagagc*

1741 *tgtttttacg atcccttcag gattacaaaa ttcaaagtgc gttgctagta ccaaccctat*

1801 *tttcattctt cgccaaaagc actctgattg acaaatacga tttatctaat ttacacgaaa*

1861 *ttgcttctgg gggcgcacct ctttcgaaag aagtcgggga agcggttgca aaacgcttcc*

1921 *atcttccagg gatacgacaa ggatatgggc tcactgagac tacatcagct attctgatta*

1981 *cacccgaggg ggatgataaa ccgggcgcgg tcggtaaagt tgttccattt tttgaagcga*

2041 *aggttgtgga tctggatacc gggaaaacgc tgggcgttaa tcagagaggc gaattatgtg*

2101 *tcagaggacc tatgattatg tccggttatg taaacaatcc ggaagcgacc aacgccttga*

2161 *ttgacaagga tggatggcta cattctggag acatagctta ctgggacgaa gacgaacact*

2221 *tcttcatagt tgaccgcttg aagtctttaa ttaaatacaa aggatatcag gtggcccccg*

2281 *ctgaattgga atcgatattg ttacaacacc ccaacatctt cgacgcgggc gtggcaggtc*

2341 *ttcccgacga tgacgccggt gaacttcccg ccgccgttgt tgttttggag cacggaaaga*

2401 *cgatgacgga aaaagagatc gtggattacg tcgccagtca agtaacaacc gcgaaaaagt*

2461 *tgcgcggagg agttgtgttt gtggacgaag taccgaaagg tcttaccgga aaactcgacg*

2521 *caagaaaaat cagagagatc ctcataaagg ccaagaaggg cggaaagtcc aaattgtaa*a

2581 atgtaactgt attcagcgat gacgaaattc ttagctattg taatactgcg atgagtggca

2641 gggcggggcg taattttttt aaggcagtta ttggtgccct taaacgcctg gttgctacgc

2701 ctgaataagt gataataagc ggatgaatgg cagaaattcg ccggatcttt gtgaaggaac

2761 cttacttctg tggtgtgaca taattggaca aactacctac agagatttaa agctctaagg

2821 taaatataaa atttttaagt gtataatgtg ttaaactact gattctaatt gtttgtgtat

2881 tttagattcc aacctatgga actgatgaat gggagcagtg gtggaatgcc tttaatgagg

2941 aaaacctgtt ttgctcagaa gaaatgccat ctagtgatga tgaggctact gctgactctc

3001 aacattctac tcctccaaaa aagaagagaa aggtagaaga ccccaaggac tttccttcag

3061 aattgctaag ttttttgagt catgctgtgt ttagtaatag aactcttgct tgctttgcta

3121 tttacaccac aaaggaaaaa gctgcactgc tatacaagaa aattatggaa aaatattctg

3181 taacctttat aagtaggcat aacagttata atcataacat actgtttttt cttactccac

3241 acaggcatag agtgtctgct attaataact atgctcaaaa attgtgtacc tttagctttt

3301 taatttgtaa aggggttaat aaggaatatt tgatgtatag tgccttgact agagatcata

3361 atcagccata ccacatttgt agaggtttta cttgctttaa aaaacctccc acacctcccc

3421 ctgaacctga aacataaaat gaatgcaatt gttgttgtta acttgtttat tgcagcttat

3481 aatggttaca aataaagcaa tagcatcaca aatttcacaa ataaagcatt tttttcactg

3541 cattctagtt gtggtttgtc caaactcatc aatgtatctt atgatgtctg gatccgtc**gt**

3601 **gaccataacc acgtcggggc tgacgcggat cggacatcac actgacgtga ggcgctctgt**

3661 **cacctctcgg gccgaacccc gtcagcaccc cgcgtcactt acaaatcacg ttcgtcatga**

3721 **cggggttttc ccctgacacg taatactcgc gtcacgtcgg gacgatataa agaggcacgg**

3781 **tgtttcgact cccgcacaca gacgacgcgc cgggcggctt cctgcggccg gccgcggtgc**

3841 **cggcggctat gatcctgtgg tctccgtcca cctgttcttt cttctggcac tggtgtctga**

3901 **tcgcagtaag tgtactctcg agccgctcca aggagtcgct ccggttgtcg tggtccagcg**

3961 **acgagtcgtc tgcgtcctcg tcgtctcgta tctgtccgtt gtcagatagc aagtccgtcc**

4021 **gtctgcccca gtaccctcgc gggttcgggg acgtgtccgg ctaccgtgtc tccagttccg**

4081 **tgtccgaatg ttacgtccag cacggggtac tcgtggccgc ctggcttgtg cgcggcaact**

4141 **tctccgatac ggccccgaga gcctacggca cctggggtaa cgagcggagc gcgacccatt**

4201 **tcaaggt**

Sequence # 2: pp28-luciferase Towne

1 **gctatcgtcg ccggaaggaa accgaccccg aaactattct gttaccctaa cactcgcctc**

61 **ccgggattgt tatgaacgct tcgtgtgccc ggtatacgat tccgggacgc cgatgggcgt**

121 **tttgatgaac ttgacgtacc tctggtatct aggcgactac ggggcgatac taaaaattta**

181 **tttcggactg ttttgcgggg cctgtgtcat cacccgatcc ctcctcctga tatgtggtta**

241 **ttatccacct cgcgaataaa atgatttgtt atgatgtcat tgtttactga aaaggaatgt**

301 **gctttcccgg catgggcccg attccgagaa atggtatgat gaatcatgtg** gtcaggcgct

361 **gttctcaacg tccatataaa cgtgggtttc** **g**gtgacgtac cgagctctta cgcgtgctag

421 ctcgaggaac gcgagacccc gtcggcacgc gagtttctgc tttcgcacga cgcggcgctc

481 ttcagggcca cgctcaagcg cgcgcgcccg ctcaagccgc ccgaaccgct gcgcgagtac

541 ctggccgatc tgctgtatct caataaggcc gagtgttcgg aagtgatcgt gtttgacgcc

601 aagcacctga gtgacgacaa cagcgacggg gacgccacga tcactattaa cgcgagtctc

661 ggcctagccg cgggcgacgc cgctggcggc ggcgctgatc accacctgcg gggcagcccg

721 ggcgattcgc cgccgccgat acctttcgag gacgaaaaca caagcttggc attcccggta

781 ctgttggtaa a*atggaagac* *gccaaaaaca taaagaaagg cccggcgcca ttctatcctc*

841 *tagaggatgg aaccgctgga gagcaactgc ataaggctat gaagagatac gccctggttc*

901 *ctggaacaat tgcttttaca gatgcacata tcgaggtgaa catcacgtac gcggaatact*

961 *tcgaaatgtc cgttcggttg gcagaagcta tgaaacgata tgggctgaat acaaatcaca*

1021 *gaatcgtcgt atgcagtgaa aactctcttc aattctttat gccggtgttg ggcgcgttat*

1081 *ttatcggagt tgcagttgcg cccgcgaacg acatttataa tgaacgtgaa ttgctcaaca*

1141 *gtatgaacat ttcgcagcct accgtagtgt ttgtttccaa aaaggggttg caaaaaattt*

1201 *tgaacgtgca aaaaaaatta ccaataatcc agaaaattat tatcatggat tctaaaacgg*

1261 *attaccaggg atttcagtcg atgtacacgt tcgtcacatc tcatctacct cccggtttta*

1321 *atgaatacga ttttgtacca gagtcctttg atcgtgacaa aacaattgca ctgataatga*

1381 *attcctctgg atctactggg ttacctaagg gtgtggccct tccgcataga actgcctgcg*

1441 *tcagattctc gcatgccaga gatcctattt ttggcaatca aatcattccg gatactgcga*

1501 *ttttaagtgt tgttccattc catcacggtt ttggaatgtt tactacactc ggatatttga*

1561 *tatgtggatt tcgagtcgtc ttaatgtata gatttgaaga agagctgttt ttacgatccc*

1621 *ttcaggatta caaaattcaa agtgcgttgc tagtaccaac cctattttca ttcttcgcca*

1681 *aaagcactct gattgacaaa tacgatttat ctaatttaca cgaaattgct tctgggggcg*

1741 *cacctctttc gaaagaagtc ggggaagcgg ttgcaaaacg cttccatctt ccagggatac*

1801 *gacaaggata tgggctcact gagactacat cagctattct gattacaccc gagggggatg*

1861 *ataaaccggg cgcggtcggt aaagttgttc cattttttga agcgaaggtt gtggatctgg*

1921 *ataccgggaa aacgctgggc gttaatcaga gaggcgaatt atgtgtcaga ggacctatga*

1981 *ttatgtccgg ttatgtaaac aatccggaag cgaccaacgc cttgattgac aaggatggat*

2041 *ggctacattc tggagacata gcttactggg acgaagacga acacttcttc atagttgacc*

2101 *gcttgaagtc tttaattaaa tacaaaggat atcaggtggc ccccgctgaa ttggaatcga*

2161 *tattgttaca acaccccaac atcttcgacg cgggcgtggc aggtcttccc gacgatgacg*

2221 *ccggtgaact tcccgccgcc gttgttgttt tggagcacgg aaagacgatg acggaaaaag*

2281 *agatcgtgga ttacgtcgcc agtcaagtaa caaccgcgaa aaagttgcgc ggaggagttg*

2341 *tgtttgtgga cgaagtaccg aaaggtctta ccggaaaact cgacgcaaga aaaatcagag*

2401 *agatcctcat aaaggccaag aagggcggaa agtccaaatt* *gtaa*aatgta actgtattca

2461 gcgatgacga aattcttagc tattgtaata ctgcgatgag tggcagggcg gggcgtaatt

2521 tttttaaggc agttattggt gcccttaaac gcctggttgc tacgcctgaa taagtgataa

2581 taagcggatg aatggcagaa attcgccgga tctttgtgaa ggaaccttac ttctgtggtg

2641 tgacataatt ggacaaacta cctacagaga tttaaagctc taaggtaaat ataaaatttt

2701 taagtgtata atgtgttaaa ctactgattc taattgtttg tgtattttag attccaacct

2761 atggaactga tgaatgggag cagtggtgga atgcctttaa tgaggaaaac ctgttttgct

2821 cagaagaaat gccatctagt gatgatgagg ctactgctga ctctcaacat tctactcctc

2881 caaaaaagaa gagaaaggta gaagacccca aggactttcc ttcagaattg ctaagttttt

2941 tgagtcatgc tgtgtttagt aatagaactc ttgcttgctt tgctatttac accacaaagg

3001 aaaaagctgc actgctatac aagaaaatta tggaaaaata ttctgtaacc tttataagta

3061 ggcataacag ttataatcat aacatactgt tttttcttac tccacacagg catagagtgt

3121 ctgctattaa taactatgct caaaaattgt gtacctttag ctttttaatt tgtaaagggg

3181 ttaataagga atatttgatg tatagtgcct tgactagaga tcataatcag ccataccaca

3241 tttgtagagg ttttacttgc tttaaaaaac ctcccacacc tccccctgaa cctgaaacat

3301 aaaatgaatg caattgttgt tgttaacttg tttattgcag cttataatgg ttacaaataa

3361 agcaatagca tcacaaattt cacaaataaa gcattttttt cactgcattc tagttgtggt

3421 ttgtccaaac tcatcaatgt atcttatgat gtctggatcc gt**cgtgacca** **taaccacgtc**

3481 **ggggctgacg cggatcggac atcacactga cgtgaggcgc tctgtcacct ctcgggccga**

3541 **accccgtcag caccccgcgt cacttacaaa tcacgttcgt catgacgggg ttttcccctg**

3601 **acacgtaata ctcgcgtcac gtcgggacga tataaagagg cacggtgttt cgactcccgc**

3661 **acacagacga cgcgccgggc ggcttcctgc ggccggccgc ggtgccggcg gctatgatcc**

3721 **tgtggtctcc gtccacctgt tctttcttct ggcactggtg tctgatcgca gtaagtgtac**

3781 **tctcgagccg ctccaaggag tcgctccggt tgtcgtggtc cagcgacgag tcgtctgcgt**

3841 **cctcgtcgtc tcgtatctgt ccgttgtcag atagcaagtc cgtccgtctg ccccagtacc**

3901 **ctcgcgggtt cggggacgtg tccggctacc gtgtctccag ttccgtgtcc gaatgttacg**

3961 **tccagcacgg ggtactcgtg gccgcctggc ttgtgcgcgg caacttctcc gatacggccc**

4021 **cgagagccta cggcacctgg ggtaacgagc ggagcgcgac ccatttcaag gt**
